# Supplementary material for: A UMLS-based spell checker for natural language processing in vaccine safety
Source: BMC Med Inform Decis Mak. 2007 Feb 12;7:3. doi: 10.1186/1472-6947-7-3 (PMC1805499; doi:10.1186/1472-6947-7-3)
Supplement: Additional file 13 — RAP application directory. Sets up directory for RAP (RDF application for PHP) source code [file 1472-6947-7-3-S13.gz › rap/doc/phpdoc/__filesource/fsource_resModel__resModelResProperty.php.html]

xml version="1.0" encoding="iso-8859-1"?


File Source for ResProperty.php


# Source for file ResProperty.php

Documentation is available at ResProperty.php

```
1. <?PHP
2. // ----------------------------------------------------------------------------------
3. // Class: ResProperty
4. // ----------------------------------------------------------------------------------


8. /**
9. * An RDF Property.
10. *
11. * <BR><BR>History:<UL>
12. * <LI>09-10-2004                : First version of this class.</LI>
13. *
14. * @version  V0.9.1
15. * @author Daniel Westphal <mail at d-westphal dot de>
16. *
17. *
18. * @package resModel
19. * @access    public
20. ***/
21. class ResProperty extends ResResource
22. {

24. /**
25. * Constructor
26. * You can supply a URI
27. *
28. * @param string $uri
29. * @access    public
30. */
31. function ResProperty($uri)
32. {
33. parent::ResResource($uri);
34. }
35. }
36. ?>
```

Documentation generated on Thu, 7 Jul 2005 13:43:46 +0200 by phpDocumentor 1.3.0RC3
